# Topiramate Induced Acute Angle Closure Glaucoma

**DOI:** 10.2174/1874364100802010046

**Published:** 2008-03-28

**Authors:** A Aminlari, M East, W Wei, D Quillen

**Affiliations:** Department of Ophthalmology, Pennsylvania State University Milton S. Hershey Medical Center, Hershey, USA

## Abstract

Topiramate is an oral sulphamate medication primarily used for seizure, migraine and neuropathic pain. It has been associated with secondary angle closure, which can mimic acute angle closure glaucoma. Suspicion for medication induced angle closure glaucoma should be higher whenever angle closure presents bilaterally. We present two cases of bilateral angle closure glaucoma secondary to topiramate.

We present two cases of bilateral secondary angle closure glaucoma related to Topiramate which mimic primary acute angle closure glaucoma. The first case is a 48 year old female referred by an outside ophthalmologist for acute vision loss and elevated intraocular pressure. The patient initially noted a “fog” of her vision in both eyes and halos around lights. Soon after noticing these symptoms, she developed a bifrontal headache. Her past ocular history was significant for convergence insufficiency and visual difficulties after suffering a concussion during a motor vehicle accident in November 2003. Her medical history was notable for bipolar disorder, depression, hypothyroidism, and chronic pain secondary to cervical disc disease. Her medications included lithium, thiothixene, dicyclomine, mirtazapine, clonazepam, bupropion, atorvastatin, lexothyroxine, and she was recently on topiramate, 2 weeks prior, for pain control. Her family history was significant for cataracts and retinal detachment, but no glaucoma.

Her vision was 20/50 in each eye with correction. Pupils were both 7mm and sluggishly reactive without a relative afferent pupillary defect. Despite aggressive topical aqueous suppressants, pilocarpine and IV mannitol, intraocular pressure (IOP) on arrival was 67 mmHg right eye and 78 mmHg left eye. Slit lamp exam revealed mild chemosis, injection and ciliary flush with diffuse stromal haze and very shallow peripheral anterior chambers with areas of iridocorneal touch in both eyes. Gonioscopy showed closed angles, and ophthalmoscopy revealed normal optic discs bilaterally, with healthy rim tissue and 0.2-0.3 cup to disc ratios in both eyes. Peripheral retinal exam and standard B-scan ultrasonography showed no apparent suprachoroidal effusion. The diagnosis of bilateral secondary acute angle closure glaucoma due to topiramate was made and the patient was managed with topical aqueous suppressants, oral hyperosmotics and discontinuing pilocarpine. Her intraocular pressure improved, but was not adequately controlled until cycloplegia was initiated. Her pain management physician was notified and the topiramate was discontinued. Over the next week, the patient’s vision returned to 20/20 in each eye. Cycloplegic retinoscopy revealed -0.75 sphere right eye and -0.25 sphere left eye. Topical aqueous suppressants and cycloplegia were continued until intraocular pressure on day 8 was 15 mmHg left eye and 16 mmHg right eye. The patient was instructed to taper her ocular medications and return to the care of her regular referring ophthalmologist.

Our second patient is a 53 year old Asian male who presented to our emergency room with severe headaches and vision loss. He had no ocular history and a past medical history significant only for cluster headaches and hyperlipidemia. Family history was not significant for ocular diseases. He had been complaining of right sided headaches and photophobia for the past 2 months, and was diagnosed with cluster headaches by his primary care physician. He was undergoing oxygen therapy at home, and his medications included eletriptan, sumatriptan, atorvastatin and topiramate. His topiramate was started 6 weeks ago by his primary care physician in an attempt to better control his headaches. A month prior to his presentation, he was evaluated by an optometrist who noted normal anterior structures and a normal IOP. On the night before his arrival to the emergency room, he woke up with severe headaches, photophobia and blurry vision in both eyes. On exam, his vision was 20/400 in both eyes, and his pupils were 6mm in both eyes and sluggishly reactive, without an appreciable relative afferent papillary defect. His IOP was 72 mmHg in the right eye and 68 mmHg in the left eye initially. He was given acetazolamide and timolol upon arrival by the emergency room, which did not significantly lower his pressure. Slit lamp exam revealed significant chemosis, mild injection and ciliary flush and very shallow peripheral anterior chambers with areas of iridocorneal touch in both eyes. Careful examination of the lens revealed glaucomflecken. Gonioscopy showed closed angles, and ophthalmoscopy revealed normal optic discs bilaterally. B scan ultrasonography showed questionable suprachoroidal effusions in both eyes. The cornea was initially clear, but progressively became more edematous during his course in the emergency room. Due to his poorly controlled IOP, he was started on multiple topical aqueous suppressants and IV mannitol, which lowered his IOP to 34 mmHg in the right eye and 37 mmHg in his left eye. The differential was bilateral secondary angle closure glaucoma due to topiramate versus primary angle closure glaucoma, for which the treatment is vastly different. The patient was started on pilocarpine so that he could undergo bilateral laser peripheral iridotomies (LPI). After undergoing bilateral LPIs, his IOP did not significantly change. He was then started on topical prednisolone for his anterior chamber inflammation and cycloplegics, the recognized treatment for secondary angle closure due to topiramate. He was maintained on topical aqueous suppressants and oral acetazolamide. Pilocarpine was stopped and the patient was also told to discontinue his topiramate. His pressure gradually normalized over the next several days, his vision improved to 20/40 bilaterally. His topical aqueous suppressants were tapered as his IOP remained stable. Gonioscopy after his IOP normalized revealed bilateral open angles and several small bilateral peripheral anterior synechiae.

Topiramate, brand name Topamax, is an anticonvulsant drug that is used for seizure, migrane and neuropathic pain. It is a sulfamate-substituted monosaccharide. Other sulfa based drugs such as acetazolamide, hydrocholorothiazide and cotrimoxazole have been associated with secondary angle closure glaucoma [1]. Topiramate was first implicated as a cause of bilateral acute angle closure glaucoma in a case report by Banta, *et al*. in July of 2001 [2]. A recent review of case reports of adverse effects of topiramate use revealed abnormal vision, acute secondary angle closure glaucoma, acute myopia and suprachoroidal effusions [3]. The proposed mechanism of myopia and secondary angle closure is choroidal effusion and forward rotation of the iris-lens diaphragm. The effusion places pressure on the vitreous body and compresses the lens-iris diaphragm, causing anterior displacement and closure of the angle [4-8]. The treatment for topiramate induced secondary angle closure are cycloplegia and topical corticosteroids. Cycloplegia relaxes the cilary body and tighten the zonules, keeping the iris-lens diaphragm in check.

Topiramate use can cause an angle closure glaucoma that presents in a variety of forms and can mimic primary acute angle closure glaucoma. A careful inquiry of current and past medications is critical in the evaluation of angle closure glaucoma. Most cases of topiramate-associated angle-closure glaucoma present within the first 2 weeks of treatment but reactions have been reported within hours of the first dose or as long as seven weeks after onset of therapy. A recent case report revealed a patient who took topiramate three times one month prior to the onset of her symptoms, did not respond to LPI for acute angle closure glaucoma, and only then was diagnosed with topiramate associated secondary angle closure, illustrating the importance of a careful medication inquisition and the difficulty of differentiating primary versus secondary angle closure [9]. Our first patient presented with pupils which were reactive, serving as a clue that this patient did not have pupillary block, and yet did not have evidence of the classic suprachoriodal effusions which were expected to be seen on B-scan. In addition, our first patient was chronically taking dicyclomine, an anticholinergic medication, for irritable bowel syndrome which could predispose patients to angle closure glaucoma. However, given the recent initiation of topiramate, and lack of response to standard treatment, it is unlikely that the patient’s glaucoma was attributable to dicyclomine. Our second patient presented with evidence pointing towards both acute angle closure glaucoma and topiramate induced secondary angle closure glaucoma. He had sluggish mid dilated pupils and glaucomflecken, but also choroidal effusions on B-scan. His IOP did not respond to standard treatment for acute angle closure glaucoma, including LPI. He only improved after aggressive cycloplegia and topical steroids. When faced with an unusual presentation of bilateral angle closure glaucoma, with a history of topiramate use being elicited on history, it is reasonable to initially treat the patient with bilateral LPIs and then topical corticosteroids and cycloplegics.
